# Successful Endoscopic Management of Fish Bone Embedded into the Bladder Wall

**DOI:** 10.1155/2010/578058

**Published:** 2010-10-26

**Authors:** Koichi Kodama, Mitsuo Ofude, Isamu Motoi, Yoshinobu Hinoue, Katsuhiko Saito

**Affiliations:** ^1^Department of Urology, Toyama City Hospital, 2-1 Imaizumihokubu-cho, Toyama, Toyama 939-8511, Japan; ^2^Department of Internal Medicine, Toyama City Hospital, 2-1 Imaizumihokubu-cho, Toyama, Toyama 939-8511, Japan; ^3^Department of Pathology, Toyama City Hospital, 2-1 Imaizumihokubu-cho, Toyama, Toyama 939-8511, Japan

## Abstract

We report a case of a pyogenous vesical abscess resulting from an ingested fish bone embedded in the bladder wall that was treated endoscopically in an asymptomatic man. Computed tomography of the abdomen showed a linear radiopaque structure in the thickened left anterolateral wall of the bladder. Cystoscopy revealed a protruding mass, covered with normal-appearing mucosa, with outflow of pus from a shallow recess. Histopathological findings indicated that the transurethrally removed linear structure, located in the submucosa, was compatible with fish bone. A high index of suspicion should be maintained for the correct diagnosis to be made.

## 1. Introduction

Foreign body (FB) ingestion is not an uncommon problem encountered in clinical practice. Most ingested FBs pass through the gastrointestinal (GI) tract uneventfully within one week, and GI perforation is rare, occurring in less than 1% of patients [1]. FB perforation occurs in all segments of the GI tract although it tends to occur in regions of acute angulation such as the ileocecal and rectosigmoid junctions [2]. Fish bones are the most commonly ingested objects and a common cause of FB perforation of the GI tract [3]. However, the development of abscess formation after the migration of a fish bone into an adjacent organ such as the bladder is extremely rare. We describe the first case of a pyogenous vesical abscess resulting from a fish bone embedded in the submucosa of the bladder treated transurethrally. 

## 2. Case Presentation

**Figure 1:**
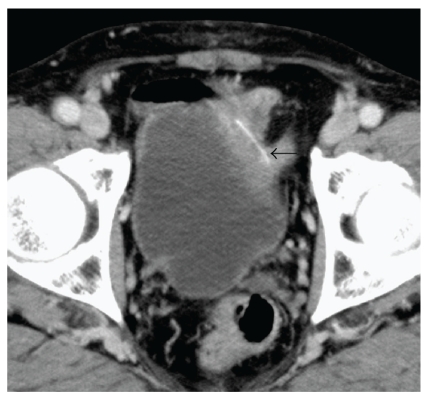
CT scan showed a linear radiopaque structure (*arrow*) traversing the thickened anterolateral wall of the bladder.

**Figure 2:**
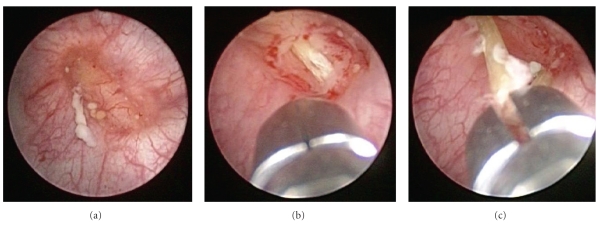
Cystoscopic view. A protruding mass with outflow of pus from a shallow recess on the top (a). A linear structure was seen after a cold cup biopsy of the mucosa was performed (b). The structure was removed using forceps (c).

A 73-year-old man with a history of hepatocellular carcinoma associated with hepatitis C virus-related cirrhosis was referred to the Department of Urology because a thickened wall of the urinary bladder was incidentally detected by computed tomography (CT). The patient had no apparent abdominal and voiding symptoms. He was afebrile, and physical examination findings were almost normal. Routine laboratory examinations were unremarkable; however, urinalysis demonstrated sterile pyuria. Voided urine cytology results demonstrated low-grade urothelial carcinoma. An X-ray film of the kidneys, ureters, and bladder showed no abnormality. CT of the abdomen demonstrated that a linear radiopaque structure, measuring 26 mm in length, traversed the thickened left anterolateral wall of the bladder (Figure 1). The lesion involved the subserosal part of the sigmoid colon. Cystoscopy revealed a protruding mass on the left anterolateral wall of the bladder, covered with normal-appearing mucosa, with outflow of pus from a shallow recess on the top (Figure 2(a)). Sigmoidoscopy appeared completely normal. 

**Figure 3:**
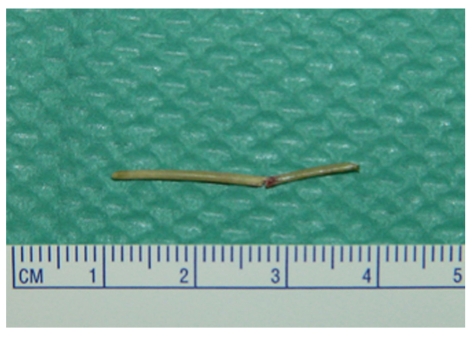
Gross examination revealed one linear, solid, and white-yellow structure, compatible with fish bone.

The patient gave a history of ingesting a fish bone accidentally. There may have been a time lag of one month from the ingestion. Clinical history and CT findings strongly suggested an abscess of the bladder wall secondary to migration of a fish bone. Transurethral biopsy of the bladder prior to partial cystectomy was performed because malignancy could not be entirely excluded. When a cold cup biopsy of the mucosa was performed, a linear structure in the submucosa was observed (Figure 2(b)) and removed transurethrally using forceps (Figure 2(c)). Gross examination revealed one linear, solid, and white-yellow structure, measuring 28 mm in length (Figure 3). Urinary leak was not detected on cystography, and his Foley catheter was removed on the seventh day after the procedure. We were concerned about complications such as peritonitis or vesicoenteric fistula resulting from the removal of the foreign body, but the postoperative course was uneventful.

Histopathological findings indicated bladder mucosa with nonspecific chronic inflammation, rich in neutrophils, without evidence of malignancy, and containing putrid skeletal bone, compatible with fish bone. This patient was thought to have a fish bone perforation of the sigmoid colon and subsequent penetration of the bladder.

At one month postoperatively, CT scan showed neither a thickened wall of the bladder nor residual fish bone. Urinalysis was normal, and voided urine cytology results were negative. In case, an artifact induced by the fish bone could have led to the false-positive diagnosis of urothelial carcinoma based on the cytology results. 

## 3. Discussion

Ingested fish bone perforation usually results in the development of peritonitis, an intraabdominal abscess, or, very rarely, after the migration of the object into an adjacent organ such as the liver [4, 5], pancreas [6], or bladder, abscess formation. To our knowledge, 22 cases of perivesical or vesical abscess formation secondary to fish bone have been described in the literature [7]. In these cases, the preoperative diagnosis of complications from fish bone ingestion was seldom made. Twelve patients had lower abdominal pain and/or irritable bladder, and 5 patients had palpable abdominal mass. The mean fish bone length was 27 mm (range 6–50 mm) including our case. The sites of perforation included the small intestine (*n* = 3), ileocecum (*n* = 2), sigmoid colon (*n* = 2), and rectum (*n* = 1). All patients except ours underwent laparotomy for management. In our patient, transurethral surgery offered the most appropriate treatment because the fish bone was located in the submucosa of the bladder. To the best of our knowledge, this is the first reported case of successful transurethral removal of an ingested fish bone.

FB perforation of the GI tract has a wide spectrum of clinical presentations, which can be acute or chronic. Fish bones are frequently ingested accidentally and forgotten. This problem is compounded because there may be a time lag of months or even years between ingestion and the onset of symptoms.

Nonmetallic FBs, such as fish bones, are rarely detected on radiographs [8]. This problem has been illustrated in the studies of fish bone ingestion showing that the degree of radiopacity of the bone depends on the species of fish [9]. CT may be useful in the correct preoperative diagnosis of FB perforation. Fish bone is visualized on CT as a linear or circumlinear calcified lesion with adjacent areas of inflammation or abscess formation. However, CT has potential limitations in the detection of FBs. Goh et al. [10] reported that the sensitivity of CT in the detection of intraabdominal fish bones was 71.4% (5/7) for initial reports but improved to 100% (7/7) on retrospective review of CT scans. The main limitation is the lack of observer awareness.

This case demonstrates an unusual presentation of fish bone migration into the bladder wall that resulted in the development of an abscess. It illustrates the difficulty in making the correct diagnosis, unless a high index of suspicion is maintained.

## References

[1] Maleki M, Evans WE (1970). Foreign-body perforation of the intestinal tract. Report of 12 cases and review of the literature. *Archives of Surgery*.

[2] Madrona AP, Hernández JAF, Prats MC, Riquelme JR, Paricio PP (2000). Intestinal perforation by foreign bodies. *European Journal of Surgery*.

[3] Lai ATY, Chow TL, Lee DTY, Kwok SPY (2003). Risk factors predicting the development of complications after foreign body ingestion. *British Journal of Surgery*.

[4] Dugger K, Lebby T, Brus M, Sahgal S, Leikin JB (1990). Hepatic abscess resulting from gastric perforation of a foreign object. *American Journal of Emergency Medicine*.

[5] Masunaga S-I, Abe M, Imura T, Asano M, Minami S, Fujisawa I (1991). Hepatic abscess secondary to a fishbone penetrating the gastric wall: CT demonstration. *Computerized Medical Imaging and Graphics*.

[6] Goh BKP, Jeyaraj P-R, Chan H-S (2004). A case of fish bone perforation of the stomach mimicking a locally advanced pancreatic carcinoma. *Digestive Diseases and Sciences*.

[7] Imamoto T, Tobe T, Mizoguchi K, Ueda T, Igarashi T, Ito H (2002). Perivesical abscess caused by migration of a fish bone from the intestinal tract. *International Journal of Urology*.

[8] Coulier B, Tancredi M-H, Ramboux A (2004). Spiral CT and multidetector-row CT diagnosis of perforation of the small intestine caused by ingested foreign bodies. *European Radiology*.

[9] Ell SR, Sprigg A (1991). The radio-opacity of fishbones—species variation. *Clinical Radiology*.

[10] Goh BKP, Tan Y-M, Lin S-E (2006). CT in the preoperative diagnosis of fish bone perforation of the gastrointestinal tract. *American Journal of Roentgenology*.

